# The Experimental Pain Experienced in a Cold Pressor Task Is Influenced by Hemoglobin Levels in Young Adult Females

**DOI:** 10.7759/cureus.65518

**Published:** 2024-07-27

**Authors:** Indu Saxena, Manoj Kumar, Nandini Srivastava, Sameer Srivastava, Aniruddha Sen, Ashish Arvind, Amar Preet Kaur

**Affiliations:** 1 Department of Biochemistry, All India Institute of Medical Sciences, Gorakhpur, Gorakhpur, IND; 2 Department of Physiology, Maharshi Vashishtha Autonomous State Medical College, Basti, Basti, IND; 3 Department of Physiology, All India Institute of Medical Sciences, Gorakhpur, Gorakhpur, IND

**Keywords:** young adult female, anemia, pain sensitivity, cardiovascular reactivity, cold pressor task, experimental pain

## Abstract

Introduction: The intensity of pain perceived for the same noxious stimulus is different in different persons, depending on the biological, psychological, and social factors related to the individual. In clinical practice, it is important to know the factors influencing pain perception. The presence of anemia may affect pain perception.

Methods: This study was conducted on 73 female subjects of whom 25 were non-anemic, 24 had mild, and 24 had moderate anemia. Experimental pain was produced by cold pressor task (CPT). Pain response was evaluated in terms of cardiovascular reactivity (CVR - changes in blood pressure and heart rate), and pain sensitivity (PS - pain threshold, pain tolerance, and pain rating).

Results: Anemic subjects showed higher CVR to stress. The average increase in systolic blood pressure was 5.28 mm of Hg in non-anemic, compared to 3.25 in mildly anemic and 9.00 mm of Hg in moderately anemic subjects. The average increase in diastolic blood pressure was 2.24 mm of Hg in non-anemic, 2.5 in mildly anemic, and 4.83 mm of Hg in moderately anemic subjects. The average increase in heart rate was 2.88 beats per minute (bpm) in non-anemic, 4.83 in mildly anemic, and 7 bpm in moderately anemic subjects. Pain rating was higher in anemic subjects (average 7.21) compared to the non-anemic subjects (average 6.44).

Conclusion: CPT-induced pain causes greater cardiovascular reactivity in anemic patients. The average pain rating is higher in anemic subjects.

## Introduction

Pain is one of the most common symptoms of disease and tissue damage [1]. It is an unpleasant sensory and emotional experience that is protective in nature as it warns the subject against harmful conditions within or outside the body. Besides being influenced by the biology of the noxious event, the magnitude of pain perceived by an individual is affected by biological, psychological, and social factors. The intensity of pain perceived by different individuals to identical stimuli can be different. The intensity of experimental pain perceived has been shown to vary with gender [2], ethnicity [3], and culture [4] of the individual. The expectation of pain can increase the intensity of the perceived pain [5,6], while a suggestion of relief can have an analgesic effect [6]. Psychological and behavioral interventions have become important in pain management. It is important to study the conditions that can aggravate or alleviate pain perception. Mental distraction may help decrease pain sensitivity (PS) [7]. Hansen et al. [8] have reviewed the significance of nocebo effects and the importance of positive expressions in clinical practice.

The presence of anemia may affect the pain response of the subject. In this study, we have compared the pain response, i.e., PS and cardiovascular reactivity (CVR), to experimental pain produced by cold pressor tasks in anemic and non-anemic subjects.

## Materials and methods

This study was initiated after obtaining ethical clearance from the Institute’s Human Ethical Committee (IHEC/AIIMS-GKP/BMR/95/2022, approval date March 21, 2022). Students of Bachelor of Medicine and Bachelor of Surgery (MBBS) and Bachelor of Science in Nursing were requested to volunteer for the study. A total of 213 students (103 female) volunteered for the study. Signed informed consent (which included a method of resting heart rate (RHR) the cold pressor task, CPT) was obtained from all subjects at the time of their reporting to volunteer.

Hemoglobin measurement

Sahli’s method [9] was used for the measurement of hemoglobin, using blood obtained from the finger prick of the subject. Only one male subject had mild anemia, while 53 female subjects were anemic; we therefore decided to conduct the study on female subjects only.

Pain perception has been shown to vary across the different phases of the menstrual cycle [10]. People may forget the date of their last period; therefore, subjects were requested to report between 24 and 48 hours (on the second day) after the initiation of the menstrual period for the CPT.

Inclusion and exclusion criteria

The inclusion criteria for the study were female subjects aged 18-25 years, while the exclusion criteria included pregnancy, lactation, history of chronic or acute illness, use of contraceptive pills, and use of analgesics within the past 48 hours. Subjects with a history of bone injury in the non-dominant limb were also excluded from the study as the non-dominant hand was immersed in cold water during the cold pressor task. In our previous study, two persons with a history of bone injury in the immersed limb had reported pain even after the removal of the hand from cold water. Subjects with resting blood pressure above 120/80 mm of Hg (pre-hypertensive or hypertensive) and RHR more than 100 beats per minute (bpm) were also excluded as pain response has been linked to resting blood pressure [11]. None of the enrolled subjects reported the use of tobacco or alcohol.

Twenty-five subjects reported taking analgesics due to period cramps in the past 48 hours and were therefore excluded from the study. Two subjects were prehypertensive (resting blood pressure between 120-139 and 80-89 mm Hg), while three subjects had RHRs higher than 100 bpm. The CPT was thus carried out on 73 female subjects aged 18-25 years, reporting between 24-48 hours after onset of menstrual period.

Categorization of subjects

The subjects were categorized into three groups: Group I, normal hemoglobin; Group II, mildly anemic; and Group III, moderately anemic. Anemia was considered as mild when the hemoglobin level was between 10 and 12 g/dL, and moderate when the hemoglobin level was between 8 and 10 g/dL. None of the volunteers had hemoglobin less than 8 g/dL (severe anemia). Subjects with hemoglobin 12 g/dL or more were considered normal [12].

Method of CPT

The experiment was carried out in a quiet air-conditioned room maintained at 24-25 degrees Celsius. The subject was seated in a comfortable chair, and RHR and blood pressure were recorded. The subject was asked to immerse her non-dominant hand, palm down, up to 5 cm above the wrist, into a circulating water bath maintained at 0-1 degree Celsius by using ice flakes in the water reservoir. She was asked to report the time when she first felt the pain and to remove her hand from the water bath whenever she felt the pain was too much to bear. Two separate stopwatches were used to record the pain threshold (time in seconds after which the subject reported feeling pain) and pain tolerance (difference between the total time of immersion and pain threshold). Blood pressure and heart rate were again recorded immediately after the CPT. The subject was asked to rate the pain perceived during CPT on a visual analog scale (VAS) [13] after the test. 

Evaluation of pain response

Pain response is evaluated here as two components: CVR and PS. Differences in the values of systolic blood pressure (ΔSBP), diastolic blood pressure (ΔDBP), and heart rate (ΔHR) measured before and immediately after the CPT are taken as CVR [14]. PS is recorded in terms of pain threshold, pain tolerance, and pain rating on the VAS.

Data analysis

Parameters of CVR and pain response obtained before and after CPT were compared by ANOVA between the three groups of subjects (normal hemoglobin, mildly anemic, and moderately anemic). Pearson correlation was checked between various parameters. Statistical Product and Service Solutions (SPSS, version 27.0.1; IBM SPSS Statistics for Windows, Armonk, NY) was used for statistical analysis. p-values less than 0.05 were considered significant.

## Results

Comparison of parameters within the three groups of subjects (non-anemic, mildly anemic, and moderately anemic) by ANOVA: Section A

Comparison of Baseline Parameters

Anthropometric parameters: There were no significant differences in age, weight, and circumference of the waist and hips between the three groups. Significant differences were recorded in height (subjects in Group I were taller), BMI, and waist-to-height ratio (WHtR) (average BMI and WHtR were lower in Group I) (Table 1).

**Table 1:** Comparison of Anthropometric Parameters Between the Three Groups of Subjects by ANOVA Group I: Hemoglobin > 12 g/dL; Group II: Hemoglobin 10 to < 12 g/dL; Group III: Hemoglobin 8 to < 10 g/dl blood. BMI: Body mass index, WHR: Waist-to-hip ratio, WHtR: Waist-to-height ratio

Parameter	Total (n = 73)		Group I (n = 25)		Group II (n= 24)		Group III (n = 24)		p-value
	Mean	SD	Mean	SD	Mean	SD	Mean	SD	
Age (years)	20.81	2.09	20.76	2.07	20.63	1.81	21.04	2.42	0.785
Weight (kg)	57.99	1.44	57.44	6.62	58.63	5.63	57.92	7.05	0.813
Height (cm)	153.07	5.78	156.36	6.24	150.33	3.91	152.38	5.36	0.001
BMI (kg/m^2^)	24.72	2.75	23.44	2.65	25.89	2.25	24.87	2.83	0.006
Waist (cm)	76.96	6.89	76.48	6.91	75.33	6.22	79.08	7.24	0.154
Hip (cm)	95.75	6.84	96.08	7.44	94.83	5.14	96.33	7.83	0.723
WHR	0.80	0.04	0.79	0.05	0.79	0.04	0.82	0.04	0.083
WHtR	0.50	0.04	0.49	0.04	0.50	0.04	0.52	0.04	0.047

Hemoglobin, resting blood pressure, and heart rate: Table 2 compares the hemoglobin, resting blood pressure, and heart rate of the subjects in the three groups. No significant differences are present in the resting blood pressure and heart rate between the three groups.

**Table 2:** Comparison of Hemoglobin Levels, Blood Pressure, and Heart Rate Amongst the Three Groups of Subjects by ANOVA Group I: Hemoglobin > 12 g/dL; Group II: Hemoglobin 10 to < 12 g/dL; Group III: Hemoglobin 8 to < 10 g/dl blood; RSBP: Resting systolic blood pressure; RDBP: Resting diastolic blood pressure; RHR: Resting heart rate

Parameter	Total (n = 73)		Group I (n = 25)		Group II (n = 24)		Group III (n = 24)		p-value
	Mean	SD	Mean	SD	Mean	SD	Mean	SD	
Hemoglobin (g/dL)	11.04	1.78	13.14	0.66	10.76	0.58	9.12	0.61	< 0.0001
RSBP (mm of Hg)	112.77	5.41	110.96	5.57	114.25	5.51	113.17	4.79	0.093
RDBP (mm of Hg)	71.73	5.429	70.32	6.421	73.33	5.459	71.58	3.821	0.150
RHR (/minute)	78.71	3.15	77.68	3.73	78.83	2.76	79.67	2.61	0.084

Comparison of Pain Response

The pain response was considered in terms of CVR (changes in blood pressure and heart rate) and PS (pain threshold, pain tolerance, and pain rating).

Table 3 compares the CVR and PS between the three groups of subjects. Significant differences are seen in the parameters of CVR: ΔSBP, ΔDBP, and ΔHR, with Group III (moderately anemic) subjects having the highest average values of the three parameters. There was no significant difference in the average values of pain threshold. Pain tolerance was the highest in Group I, while pain rating was higher in Groups II and III.

**Table 3:** Comparison of Pain Response Components (CVR and PS) Amongst the Three Groups of Subjects by ANOVA CVR: Cardiovascular reactivity; ΔSBP: Change in systolic blood pressure (mm of Hg); ΔDBP: Change in diastolic blood pressure (mm of Hg); ΔHR: Change in heart rate (beats per minute); PS: Pain sensitivity; P Th: Pain threshold (seconds); P Tol: Pain tolerance (seconds); PR: Pain rating (on the visual analog scale, VAS)

		Total (n = 73)		Group I (n = 25)		Group II (n = 24)		Group III (n = 24)		p-value
		Mean	SD	Mean	SD	Mean	SD	Mean	SD	
CVR	ΔSBP	5.84	4.99	5.28	4.43	3.25	2.75	9.00	5.66	< 0.0001
	ΔDBP	3.18	3.03	2.24	3.13	2.50	1.80	4.83	3.33	0.004
	ΔHR	4.88	4.48	2.88	4.55	4.83	3.12	7.00	4.76	0.004
PS	P Th	18.93	8.97	17.88	8.42	19.17	9.43	19.79	9.32	0.753
	P Tol	30.64	18.38	38.76	20.27	21.21	10.49	31.63	18.79	0.003
	PR	6.95	0.88	6.44	0.87	7.21	0.83	7.21	0.72	0.001

Pearson correlation of hemoglobin and resting cardiovascular parameters with anthropometric parameters: Section B

Table 4 shows the correlation of hemoglobin, blood pressure, and heart rate with the anthropometric parameters. Weak but significant positive correlation is present between Hb level and height. Moderate positive correlation is present between resting systolic blood pressure (RSBP) and weight, BMI, waist, hips, and waist-to-height ratio (WHtR). A moderate positive correlation is present between resting diastolic blood pressure (RDBP) and weight, BMI, waist, and WHtR. A weak positive correlation is present between RHR and waist, hips, and WHtR. RSBP, RDBP, and RHR are positively correlated with each other. A weak negative correlation can be seen between hemoglobin and BMI, WHtR, RSBP, and RHR.

**Table 4:** Pearson Correlation of Hemoglobin, Blood Pressure, and Heart Rate with Anthropometric Parameters Hb: Hemoglobin (g/dL); RSBP: Resting systolic blood pressure (mm of Hg); DSBP: Resting diastolic blood pressure (mm of Hg); RHR: Resting heart rate (beats/min, bpm); Weight (kg); Height (cm); BMI (kg/m^2^); Waist (cm); Hip (cm); WHR: Waist-to-hip ratio; WHtR: Waist-to-height ratio; r: Pearson coefficient; p: Significance (p-value)

		Weight	Height	BMI	Waist	Hip	WHR	WHtR	Hb	RSBP	RDBP	RHR
Hb	r	-0.085	0.309	- 0.283	- 0.187	- 0.062	- 0.226	- 0.329	1	-0.265	-0.176	-0.363
	p	0.473	0.007	0.015	0.110	0.599	0.053	0.004		0.023	0.133	0.001
RSBP	r	0.472	- 0.177	0.588	0.466	0.447	0.188	0.570	-0.265	1	0.698	0.425
	p	<0.001	0.131	<0.001	<0.001	<0.001	0.109	< 0.001	0.023		<0.001	<0.001
RDBP	r	0.437	- 0.208	0.583	0.414	0.294	0.306	0.530	-0.176	0.698	1	0.261
	p	<0.001	0.075	<0.001	<0.001	0.011	0.008	<0.001	0.133	<0.001		0.025
RHR	r	0.137	- 0.087	0.155	0.310	0.336	0.076	0.353	-0.363	0.425	0.261	1
	p	0.245	0.462	0.186	0.007	0.003	0.517	0.002	0.001	<0.001	0.025	

Pearson correlation of pain response with baseline parameters: Section C

Correlation of Pain Response with Anthropometric Parameters

Table 5 correlates the pain response parameters (CVS and PS) with the anthropometric parameters. ΔHR shows a weak positive correlation with BMI and WHtR; pain tolerance (PTol) shows weak positive correlation with height, BMI, and WHtR.

**Table 5:** Correlation of Pain Response Parameters with Anthropometric Parameters ΔSBP: Change in systolic blood pressure (mm of Hg); ΔDBP: Change in diastolic blood pressure (mm of Hg); ΔHR: Change in heart rate (beats per minute); P Th: Pain threshold (seconds); P Tol: Pain tolerance (seconds); PR: Pain rating (on the visual analog scale, VAS); WHR: Waist-to-hip ratio; WHtR: Waist-to-height ratio

		Weight (kg)	Height (cm)	BMI (kg/m^2)^	Waist (cm)	Hip (cm)	WHR	WHtR
ΔSBP	r	- 0.052	0.116	- 0.113	0.098	0.026	0.119	0.053
	p	0.661	0.326	0.337	0.408	0.828	0.314	0.655
ΔDBP	r	- 0.019	0.094	- 0.079	- 0.001	0.016	- 0.018	- 0.035
	p	0.875	0.428	0.502	0.995	0.895	0.877	0.770
ΔHR	r	0.203	- 0.166	0.315	0.228	0.183	0.127	0.316
	p	0.082	0.158	0.006	0.051	0.118	0.280	0.006
P Th	r	0.125	0.167	0.012	0.095	- 0.005	0.156	0.016
	p	0.290	0.156	0.922	0.423	0.970	0.186	0.890
P Tol	r	- 0.107	0.238	- 0.272	- 0.143	- 0.116	- 0.080	- 0.268
	p	0.365	0.042	0.019	0.223	0.327	0.500	0.021
PR	r	- 0.021	- 0.179	0.078	0.041	- 0.034	0.108	0.126
	p	0.859	0.128	0.510	0.726	0.772	0.359	0.285

Correlation of Pain Response with Hemoglobin and Resting Cardiovascular Parameters

The parameters of CVS (ΔSBP, ΔDBP, and ΔHR) and pain rating (PR) are negatively correlated with the level of hemoglobin (Table 6).

**Table 6:** Correlation of Pain Response Parameters with Hemoglobin, Blood Pressure, and Heart Rate ΔSBP: Change in systolic blood pressure (mm of Hg); ΔDBP: Change in diastolic blood pressure (mm of Hg); ΔHR: Change in heart rate (beats per minute); P Th: Pain threshold (seconds); P Tol: Pain tolerance (seconds); PR: Pain rating (on the visual analog scale, VAS); RSBP: Resting systolic blood pressure; RDBP: Resting diastolic blood pressure; RHR: Resting heart rate

		Hemoglobin (g/dL)	RSBP (mm of Hg)	RDBP (mm of Hg)	RHR (per minute)
Δ SBP	r	- 0.273	- 0.121	0.003	0.042
	p	0.019	0.306	0.983	0.723
Δ DBP	r	- 0.310	- 0.013	- 0.153	0.056
	p	0.007	0.909	0.193	0.638
Δ HR	r	- 0.423	0.134	0.142	- 0.059
	p	< 0.001	0.256	0.226	0.618
P Th	r	- 0.100	0.017	0.035	0.201
	p	0.395	0.887	0.765	0.086
P Tol	r	0.221	- 0.175	- 0.059	- 0.116
	p	0.059	0.137	0.616	0.325
PR	r	- 0.374	0.050	0.084	0.094
	p	0.001	0.670	0.476	0.426

Correlation of Parameters of Pain Sensitivity with Those of Cardiovascular Reactivity

Correlation analysis of pain sensitivity with CVR shows a weak positive correlation between pain rating and heart rate (Table 7).

**Table 7:** Correlation of Pain Sensitivity Parameters with Cardiovascular Reactivity Parameters ΔSBP: Change in systolic blood pressure (mm of Hg); ΔDBP: Change in diastolic blood pressure (mm of Hg); ΔHR: Change in heart rate (beats per minute); P Th: Pain threshold (seconds); P Tol: Pain tolerance (seconds); PR: Pain rating (on the visual analog scale, VAS)

		ΔSBP	ΔDBP	ΔHR
P Th (s)	r	0.206	0.046	- 0.030
	p	0.079	0.694	0.801
P Tol (s)	r	0.013	0.024	- 0.200
	p	0.911	0.842	0.088
PR (VAS)	r	0.111	0.181	0.322
	p	0.348	0.123	0.005

## Discussion

A comparison of baseline anthropometric parameters by ANOVA is summarized in Table 1. The inclusion criteria of our study specified age between 18 and 25 years; therefore, no significant difference was observed in the average age of the three groups.

Chronic mild/moderate anemia may be accompanied by other nutritional deficiencies during the period of growth, resulting in shorter height of the anemic subjects (Groups II and III, Table 1).

Although hemoglobin levels have been shown to be positively correlated with weight and BMI [15-17], in our study, subjects with mild/moderate anemia had higher average BMI and WHtR. Stoffel et al. [18] have shown that young women with central obesity have higher serum hepcidin levels, reducing iron absorption in the gut. The anemic subjects in our study had central obesity as apparent from the higher values of WHtR (Table 1). This is also apparent from the weak negative correlation between Hb and WHtR (Table 4).

Table 2 compares hemoglobin and resting cardiovascular parameters by ANOVA. Since the subjects were categorized into three groups on the basis of their blood hemoglobin levels, highly significant differences were obtained in the average hemoglobin levels of the three groups.

We had selected normotensive subjects, and there was no significant difference in the blood pressure or heart rate between the subjects of the three groups (Table 2). 

The objective of our study was to compare the pain response between anemic and non-anemic subjects. A comparison of pain response between subjects of the three groups by ANOVA (summarized in Table 3) showed higher average values of CVR and pain rating in the anemic subjects. The average pain threshold was 17.88 s in Group I, 19.17 s in Group II, and 19.79 s in Group III. This difference was not significant. Average pain tolerance was 38.76 s in Group I, 21.21 s in Group II, and 31.63 s in Group III. Since the average pain tolerance was higher in subjects with normal hemoglobin values and also in those with moderate anemia, the results appear confusing and may be due to outliers. Higher pain ratings in the anemic subjects could indicate greater cold-induced vasoconstriction in these subjects leading to a greater perception of pain [19,20].

Pearson correlation of hemoglobin and resting cardiovascular parameters with anthropometric parameters showed a positive correlation between hemoglobin levels and height (Table 4) and has been discussed above. Nutritional deficiencies accompanying anemia in the growing years of the subject may be responsible for the failure to attain maximum potential height. Although only normotensive volunteers were selected to participate in this study, a moderate positive correlation can be seen between RSBP and weight, BMI, waist, and WHtR. This is not unexpected as a positive relationship between body weight and blood pressure has been recognized for a long time [21]. This correlation would probably have been stronger if the inclusion criteria had not specified normotensive subjects.

Pain response and baseline anthropometric parameters showed a significant but weak positive Pearson correlation between the change in heart rate (ΔHR) and BMI and WHtR (Table 5), indicating that pain produced by CPT has a larger effect on heart rate in persons with central adiposity. Similarly, pain tolerance is weakly correlated to height, BMI, and WHtR. Chronic pain is reportedly higher in subjects with obesity [22]. However, Emerson et al. [23] conducted a study on 38 obese and 41 non-obese subjects and have shown that pain sensitivity is not affected by BMI. Since the anemic subjects in our study had higher average BMI and WHtR, the positive correlation between P Tol and these parameters could be due to differences in hemoglobin levels.

Pain response and hemoglobin and resting cardiovascular parameters showed a weak negative correlation of CVR with hemoglobin levels (Table 6); that is, anemic subjects had higher CVR (also apparent from Table 3). Pain rating is negatively correlated with hemoglobin level, indicating that, though anemic subjects had no significant differences in pain threshold and pain tolerance, they experienced greater pain, which had a greater effect on their blood pressure and heart rate, compared to that in subjects with normal hemoglobin levels. Pain response has been shown to be affected by resting blood pressure [11]; however, this factor was absent in our study because of our exclusion criteria.

Cardiovascular reactivity and pain sensitivity showed a weak positive correlation between pain rating and heart rate (Table 7), signifying that the greater the pain perceived by the subject (pain rating), the greater the change in the heart rate.

This study shows that cardiovascular reactivity and pain rating are higher in anemic subjects exposed to CPT-induced pain.

Kasage et al. [24] and Mathews et al. [25] have shown that higher cardiovascular reactivity to stress predicts future hypertension. This may also have consequences in the case of chronic pain, when higher cardiovascular reactivity may adversely affect the body. It is possible that anemic subjects with chronic pain or suffering from frequent pain episodes may have a higher risk of future hypertension; however, a separate study is needed to prove this.

Limitations of the study

This study was performed exclusively on female subjects. Since pain response shows gender variation, a larger study including both sexes is required.

## Conclusions

Subjects with mild or moderate anemia showed greater cardiovascular reactivity and pain rating. Exposure to CPT produces vasoconstriction, which causes greater discomfort in anemic subjects, resulting in larger increase in heart rate. There is a need to investigate the effect of pain in anemic subjects produced by different stimuli. Higher CVR to pain has been linked to future hypertension; therefore, anemic patients with chronic pain or suffering frequent pain episodes could have a higher risk of future hypertension.

This study also showed a slight positive correlation between blood hemoglobin levels and height. This suggests that chronic anemia may be accompanied with other nutritional deficiencies and results in failure to attain maximum growth or stunting. A slight negative correlation between hemoglobin levels and central obesity was observed in our study. Central obesity is known to be associated with elevated hepcidin levels, which may cause impaired iron absorption and iron-deficiency anemia. Therefore, the presence of central obesity or higher than normal BMI in young adult females does not reflect absence of anemia.
